# Down-Regulatory Effects of miR-211 on Long Non-Coding RNA *SOX2OT* and *SOX2* Genes in Esophageal Squamous Cell Carcinoma 

**DOI:** 10.22074/cellj.2016.3811

**Published:** 2016-01-17

**Authors:** Mohammad Shafiee, Seyed Ahmad Aleyasin, Mohammad Vasei, Shahriar Semnani Semnani, Seyed Javad Mowla

**Affiliations:** 1 Department of Medical Genetics, National Institute of Genetic Engineering and Biotechnology, Tehran, Iran; 2 Stem Cell Research Center, Golestan University of Medical Sciences, Gorgan, Iran; 3 Department of Pathology and Digestion Disease Research Institute, Shariati Hospital, Tehran University of Medical Sciences, Tehran, Iran; 4 Golestan Research Center of Gastroenterology and Hepatology, Golestan University of Medical Sciences, Gorgan, Iran; 5 Department of Molecular Genetics, Faculty of Biological Sciences, Tarbiat Modares University, Tehran, Iran

**Keywords:** lncRNA, miR-211, SOX2, Pluripotency, Stem Cell

## Abstract

**Objective:**

MicroRNAs (miRNAs) are a class of non-coding RNAs (ncRNAs) that tran- scriptionally or post-transcriptionally regulate gene expression through degradation of their mRNA targets and/or translational suppression. However, there are a few reports on miRNA-mediated expression regulation of long ncRNAs (lncRNAs). We have previ- ously reported a significant upregulation of the lncRNA *SOX2OT* and its intronic cod- ing gene, *SOX2*, in esophageal squamous cell carcinoma (ESCC) tissue samples. In this study, we aimed to evaluate the effect of induced overexpression of miR-211 on *SOX2OT* and *SOX2* expression *in vitro*.

**Materials and Methods:**

In this experimental study, we performed both bioinformatic and experimental analyses to examine whether these transcripts are regulated by miRNAs. From the list of potential candidate miRNAs, miR-211 was found to have complementary sequences to SOX2OT and SOX2 transcripts. To validate our finding experimentally, we transfected the NT-2 pluripotent cell line (an embryonal carcinoma stem cell) with an expression vector overexpressing miR-211. The expression chang- es of *miR-211, SOX2OT*, and *SOX2* were then quantified by a real-time polymerase chain reaction (RT-PCR) approach.

**Results:**

Compared with mock-transfected cells, overexpression of *miR-211* caused a significant down-regulation of both genes (P<0.05). Furthermore, flow-cytometry analysis revealed a significant elevation in sub-G1 cell population following ectopic expression of miR-211 in NT-2 cells.

**Conclusion:**

We report here, for the first time, the down-regulation of *SOX2OT* and *SOX2* genes by an miRNA. Considering the vital role of *SOX2OT* and *SOX2* genes in pluripotency and tumorigenesis, our data suggest an important and inhibitory role for miR-211 in the aforementioned processes.

## Introduction

Long non-coding RNAs (lncRNAs) represent a large fraction of the human transcriptome, and are recently acknowledged as a new class of gene expression regulators (1,2). These molecules are found to be involved in epigenetic events as well as in regulation of pluripotency and differentiation processes (3,4). LncRNAs have been also implicated in tumorigenesis, with some classified as oncogenes or tumorsuppressor genes (5). They have also been reported to possess an enhancer-like function in human cells (6). The human genome contains thousands of lncRNAs that are expressed at low levels and in a tissue-specific manner. Compared with protein-coding genes, lncRNAs are less conserved, and about a third of them are primate-specific (7). While the expression and function of small ncRNAs [e.g. microRNAs (miRNAs)] are widely studied, much less studies have been devoted to lncRNAs. 

*SOX2* gene, encoding a master regulator of pluripotency, lies within the third intron of a lncRNA, *SOX2OT*, which is transcribed in the same direction as *SOX2*. We recently reported that *SOX2OT* is upregulated along with master regulators of pluripotency, *SOX2* and *OCT4*, in esophageal squamous cell carcinoma (ESCC). Moreover, the novel variants of *SOX2OT (SOX2OT-S1* and *SOX2OT-S2)* showed distinct expression patterns and were downregulated during the process of neural differentiation of human embryonal carcinoma stem cells known as the NTERA-2/NT-2 cell line (8,9). 

miRNAs are a class of small (18-22 nt) ncRNAs that regulate gene expression mostly at the post-transcriptional level. They contribute to various cellular processes such as cell proliferation, cell growth and development, cellular stress response and apoptosis (10). Alterations in the expression of miRNAs have been widely reported in numerous diseases including almost all types of cancers. Acting as oncogenes (oncomiRs) or tumor suppressors, miRNAs play prominent roles in cancer-related processes such as proliferation, apoptosis, metastasis and angiogenesis (11). Due to their high stability and celland tissue-specific expression patterns, miRNAs have received tremendous attention as potential diagnostic, prognostic and therapeutic agents over the past decade (12). 

*mir-211* is mapped to a frequently altered locus in cancers on chromosome 15q13. Despite its hot spot location, the exact role of miR211 in carcinogenesis has not yet been clearly defined. We used bioinformatics approaches to find potential miRNAs capable of hitting *SOX2OT* and/or *SOX2* transcripts. We then experimentally validated the down-regulation of *SOX2OT* and *SOX2* by overexpressing mir-211 in NT-2 cells. 

## Materials and Methods

### Cell culture

In this experimental study, human embryonal carcinoma stem cells (NT-2), which highly express *SOX2OT* and *SOX2* genes, were kindly provided by Dr. Peter W. Andrews at University of Sheffield, UK. Cells were cultured in Dulbecco’s Modified Eagle’s Medium (MDEM)/F12 medium (Invitrogen, USA) supplemented with 10% fetal bovine serum (FBS, Invitrogen, USA) and 100 U/ml penicillin/streptomycin (Sigma, USA) and incubated in a humidified incubator in an atmosphere of 5% CO_2_ at 37˚C.

### Bioinformatics analysis

The bioinformatics tool miRcode (http://www.mircode.org/mircode; miRcode 11, accessed June 2012) was employed to find complementary sequences of miR-211 with SOX2OT and SOX2 transcripts. miRcode is a comprehensive search tool for putative miRNA target sites across the complete GENCODE annotated transcriptome which includes 10,419 lncRNA genes in the current version. 

### mir-211 cloning in an expression vector

The recombinant expression plasmid pEGFP-C1 containing the miR-211 precursor as well as the mock vector with no insert was purchased from ParsGenome Company (Tehran, Iran). Both constructs contained Neomycin and GFP to enable selection and detection of transfected cells. PEGFPC1-miR-211 vector containing EcoR1 and BamHI restriction sites on their respective 5´ and 3´ ends of *mir-211* were used to amplify a 181 bp segment containing the pre-miR-211 sequence by specific primers (Table 1). 

### Ectopic expression of miR-211 in NT-2 cells

The NT-2 cells were seeded at a concentration of 4×10^4^ cells per well in 12-well plates and incubated for 24 hours in culture medium. The cells were transfected with 1.5 μg of pEGFP-C1-miR-211 or mock vectors, using Lipofectamin 2000 reagent (Invitrogen, USA) and according to the manufacturer’s instructions.

### RNA extraction

Cells were harvested 48 hours after transfection and total RNA was extracted from the cells using Trizol (Invitrogen, USA) according to the manufacturer’s instructions. The precipitated RNA was re-suspended in 20-30 µl RNase-free dH_2_O and was treated with DNaseI (Sigma, USA) to remove any potential trace of DNA contamination. The quality and quantity of the total RNA were then determined using agarose gel electrophoresis and spectrophotometry (measuring absorbance at 260 nm, NanoDrop2000c, Thermo Fisher Scientific Inc., Wilmington, DE, USA) respectively. 

### Synthesis of cDNA

The first strand of cDNA was synthesized by using a reverse transcriptase (RT, Takara, Japan), oligo-dT and random hexamer primers (Takara, Japan) according to the manufacturer’s instructions. For each sample, a noRT control was used in parallel to the DNase-treated RNA, to detect any potential non-specific amplification of genomic DNA. Synthesis of cDNA for expression analysis of miR-211 was performed separately by a different procedure using a miRNA amplification kit (ParsGenome, Iran). This was carried out in three main steps including addition of polyA tail, specific cDNA synthesis, and real-time PCR amplification, according to the manufacturer’s instructions.

**Table 1:** Sequence of primers used for cloning and/or amplification of all genes


Genes	Sequence of primers

*miR-211 precursor*	F:5ˊCCGGAATTCCGGTTTTACAACACCCCATTTCACC3ˊ
	R:5ˊCGCGGATCCGCGCGAGCAACAGAGTAGAACAGG3ˊ
*SOX2^a^*	F:5ˊAAGAGAACACCAATCCCATCC3ˊ
	R:5ˊTCCAGATCTATACAAGGTCCATTC3ˊ
*SOX2OT^b^*	F:5ˊGATCACCTATTATAATTTTACC3ˊ
	R:5ˊGAAACCTGTCAGGCTTTCTTC3ˊ
*GAPDH^c^*	F:5ˊGCCACATCGCTCAGACAC3ˊ
	R:5ˊGGCAACAATATCCACTTTACCAG3ˊ


^a^; *SOX2*, SRY (sex determining region Y)-box 2, ^b^; *SOX2OT, SOX2* overlapping transcript and ^c^; GAPDH, Glyceraldehyde 3-phosphate dehydrogenase.

### Quantitative polymerase chain reaction (qRT-PCR)

Expression of *mir-211*, *SOX2OT* and *SOX2* were quantified in NT2 cells transfected with pEGFP-C1-miR-211 or pEGFP-C1-mock vectors 48 hours after transfection, using ParsGenome’s miRNA amplification Kit. All experiments were performed at least in duplicate using a Bio-Rad thermal cycler (Hercules, USA) with the following conditions: 95˚C for 10 seconds, 62˚C for 10 seconds, and 72˚C for 30 seconds. Melting curves were then determined with temperature ranging 60 to 95˚C. miR-16 was chosen as an internal control (13) for normalization of miR-211 quantification. PCR efficiency for miR-211 and miR-16 was measured using standard curves generated by serial dilutions of generated cDNA. 

Specific real-time PCR primers were also designed for *SOX2OT, SOX2* and glyceraldehyde 3-phosphate dehydrogenase (GAPDH, as an internal control) using AlleleID 6.0 (Primer BioSoft, USA) and Gene Runner (version 3.02, Hastings Software Inc.) softwares. Primer sequences for amplifying cDNA of *SOX2, SOX2OT* and *GAPDH* have been listed in table 1 (length of products: 162, 128 and 115 bp, respectively). TaKaRa SYBR Premix Ex Taq ІІ master mix (2X), supplemented with ROX reference Dye II, were used for all real-time PCR reactions. To compensate for variation in the amount of input RNA and the efficacy of reverse transcriptase, GAPDH mRNA was also quantified as an internal control, and related expressions of *SOX2* and *SOX2OT* were normalized to the expression value of the internal control. All real-time PCR reactions were carried out by an ABI 7500 realtime PCR instrument (Applied Biosystems, CA) using the following cycling conditions: initiation at 94˚C for 1 minutes, amplification for 40 cycles with denaturation at 94˚C for 10 seconds, annealing at 62˚C for 10 seconds and extension at 72˚C for 40 seconds. The expression of the genes were defined based on the threshold cycle (C_t_) and relative expression levels were determined as 2-^[Ct of target gene)-(Ct of internal control)]^.

### Cell cycle analysis by flow cytometry

For cell cycle analysis, cells were collected 48 hours after transfection with pEGFP-C1miR-211 or pEGFP-C1-mock vector. The cells were washed in phosphate bovine serum (PBS) and trypsinized with 0.025% trypsin-EDTA to yield single cell suspension. Cells were then fixed in ice-cold 70% ethanol and stained with 50 μg/ml propidium iodide (PI) solution containing 10 μg/ml RNaseA (Takara, Japan) and 0.1% Triton X-100. Two groups of cells were used for flow cytometric analysis using Attune Acoustic Focusing Cytometer (Applied Biosystems, USA). Experiments were repeated at least twice and cell cycle profiles were analyzed using Flowing Software version 2.5. 

### Statistical analysis

Data are presented as mean ± SD based on a minimum of three separate experiments. Significance of differential expression was analyzed using the Student’s t test. Real-Time RT-PCR data were adjusted based on the exact PCR efficiency. SPSS software version 17.0 (SPSS, Inc., USA) was used for performing all the analyses. P<0.05 was considered statistically significant. 

## Results

### miR-211 has binding sites on SOX2OT and SOX2 transcripts

**Fig.1:**
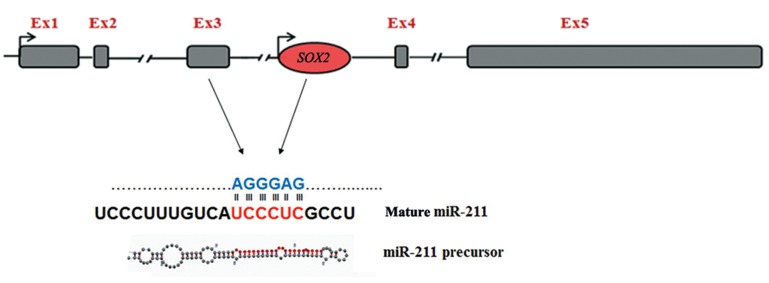
A schematic representation of the bioinformatics analysis on miR-211 potential targeting sites on *SOX2OT* and *SOX2* transcripts. miR-211 has a putative target site on the third exon of *SOX2OT*, as well as similar target sites on the *SOX2* transcript. The *SOX2OT* gene contains five exons where the intron-less gene *SOX2* embedded within its third intron. Seed sequence of the mature form of miR-211 has been represented with red font.

Bioinformatics analysis revealed a miR-211 putative target site (AGGGAG) on the third exon of *SOX2OT* at nucleotide position 659 (Fig .1). Interestingly, the same target site was also found on *SOX2* gene. 

### Induced ectopic expression of miR-211 in NT2 cells

**Fig.2:**
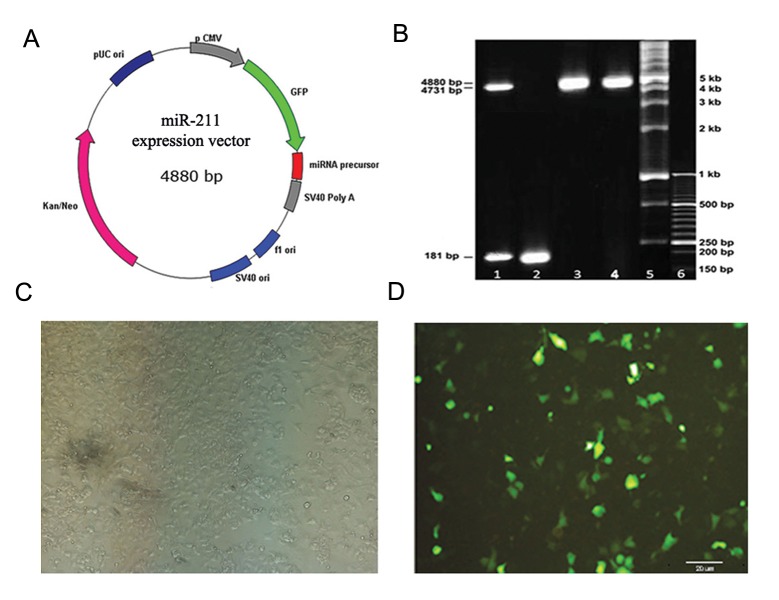
A. Recombinant vector of pEGFP-C1 containing the precursor of miR-211, B. Verifying the accuracy of cloned miR-211 precursor within pEGFP-C1-miR-211 vector by restriction digestion and PCR analysis, lane 1: Double-digest of pEGFP-C1-miR-211 with EcoRI and BamHI produced a 181 bp segment corresponding to the miR-211 precursor insert and a 4731 bp linear vector, lane 2: A 181 bp PCR segment (bottom band) is amplified by specific primers, lane 3: A single digestion of pEGFP-C1-miR-211 by EcoRI produced a 4880 bp fragment, lane 4: Single digestion of pEGFP-C1-mock digested with EcoRI produced a 4731 bp-fragment, lanes 5 and 6: 1 kb and 50 bp DNA ladders respectively, C. Phase contrast micrograph of NT-2 cells 48 hours after transfection with pEGFP-C1-miR-211 and D. Fluorescence micrograph showing transfected cells expressing GFP as a reporter. PCR; Polymerase chain reaction.

To experimentally validate the aforementioned bioinformatics results, we chose NT2 as a pluripotent cell line previously shown by us (8) to higly express *SOX2OT* and *SOX2*. Accordingly, miR211 was cloned in a pEGFP-C1 plasmid and the resultant recombinant plasmid was then employed to transiently transfect NT-2 cells (Fig .2). The real-time PCR approach was then used to measure the level of miR-211 in NT2 cells transfected with pEGFP-C1-miR-211, in comparison with the pEGFP-C1-mock transfected cells as control. A significant upregulation was observed in the cells over-expressing miR-211 (15.5-fold), 48 hours after transfection (P<0.05). 

### Over-expression of miR-211 caused down-regulation of SOX2 and SOX2OT

**Fig.3:**
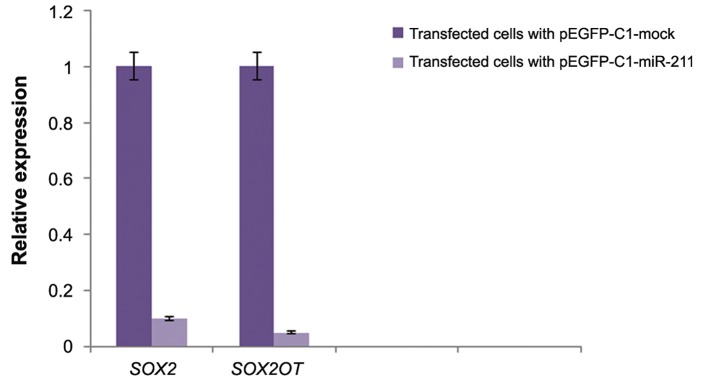
Relative expression of *SOX2* and *SOX2OT* in NT-2 cells transfected with pEGFP-C1-miR-211 or pEGFP-C1-mock (control). Ectopic expression of miR-211 precursor reduced the level of *SOX2* and *SOX2OT* transcripts in transfected cells drastically (P<0.05).

 The relative expression level of *SOX2* and *SOX2OT* was measured by real-time RT-PCR in cells transfected with pEGFP-C1-miR-211 compared with control cells. As shown in figure 3, the expression of *SOX2OT* was significantly diminished (27-fold) in cells overexpressing miR-211. Similarly, a 9-fold downregulation of *SOX2* was observed in the same cells transfected with pEGFP-C1-miR-211 (P<0.05, Fig .3). 

### Effects of miR-211 over-expression on the cell cycle progression in NT-2 cells

**Fig.4:**
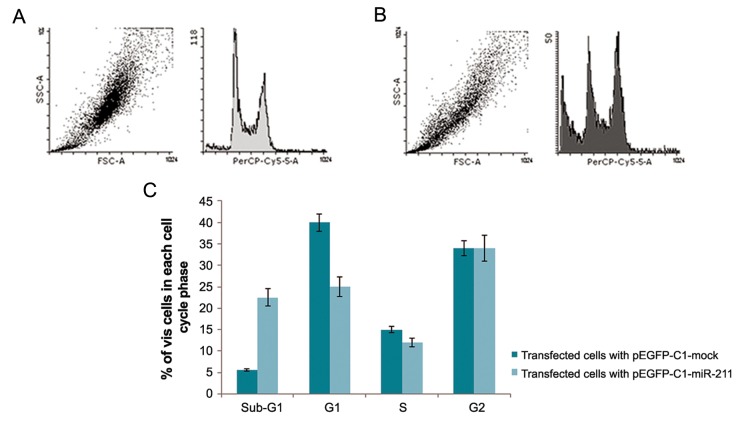
A, B. Flow cytometry data demonstrating altered cell cycle distribution of NT-2 cells following ectopic expression of miR-211 and C. Flow cytometry analysis of NT-2 cells 48 hours after transfection of cells with pEGFP-C1-miR-211 or pEGFP-C1-mock (control) revealed a significant elevation in sub-G1 cell population, suggesting increased number of cell death following ectopic expression of miR-211.

NT-2 cells were analyzed by flow cytometry cell cycle analysis 48 hours after transfection. An altered cell cycle distribution was observed in cells overexpressing *mir-211* when compared with cells transfected with the pEGFP-C1-mock vector as control. miR211 caused a significant decrease in the percentage of cells in the S and G0/G1 phases of the cell cycle. Moreover, although a significant arrest at sub-G1 was observed in cells overexpressing miR-211, no significant decrease in the percentage of the cells in G2/M phase was observed (Fig .4). 

## Discussion

lncRNAs have been reported to play an important part in stem cell biology as well as in tumor initiation and progression (14). *SOX2OT*, a dynamically regulated lncRNA, has conserved functions during vertebrate development. We recently had reported differential expression and alternative splicing of *SOX2OT* during neural differentiation of pluripotent cells and esophagus tumorigenesis (8). Here, we expanded our previous findings by exploring a potential miRNA capable of regulating *SOX2OT*. 

Transcription pattern of *SOX2OT* and *SOX2* has been proposed to be regulated in the same manner as in pluripotent stem cells such as NT2 cells (8). In this study, down-regulation of *SOX2OT* and *SOX2* have been demonstrated simultaneously with miR211 ectopic expression in NT2 cells. This finding is in agreement with the hypothesis proposed by Amaral et al. (9) and Shahryari et al. (8) on the possible role of *SOX2OT* in regulating *SOX2* expression. Silencing *SOX2OT* by specific siRNAs as reported by Shahryari et al. demonstrated an inhibitory effect on *SOX2* expression as well as an inductive one on subG1/G1 cell cycle arrest in NT2 cells (8). 

Moreover, our flow cytometry data on miR-211 over-expressing cells is in agreement with two independent studies reporting that *SOX2OT* suppression could attenuate S-phase entry and accumulation of the cells in the G0/G1 cell cycle phase (15,17). Considering the nuclear localization of *SOX2OT*, it can be speculated that this lncRNA is acting at the transcriptional level rather than posttranscriptional to regulate *SOX2* expression (8). According to our data, miR-211 also contributes to this regulatory process probably via its capacity to alter gene expression at both transcriptional and post-transcriptional levels. 

miR-211 is also thought to contribute to the cellular response to endoplasmic reticulum (ER) stress in a PKR-like endoplasmic reticulum kinase (PERK)-dependent fashion (17). This property of miR-211 may also be inferred from our current finding of significant accumulation of human NT2 cells in the sub-G1 phase of cell cycle. PERK activation is related to the cellular inhibition of protein translation (18,20) with respect to miR-211 function. In addition, one study reported that a relevant target for miR-211 is the pro-apoptotic transcription factor *chop* (17). Based on an unbiased bioinformatics search, the *chop* gene promoter has two high-relevance complementary sequences matching the seed sequence of miR-211. In this model, miR-211 interacts directly with the *chop* promoter and confers its pro-survival influence by antagonizing pro-apoptotic *chop* expression. Thus, our bioinformatics data supports a model wherein miR-211 functions in the context of ER stress signaling to regulate post-transcriptional gene silencing via a chromatin-modification-dependent mechanism to moderate induction of pro-apoptotic *chop* and potentiate cell survival. Accordingly, the transient accumulation of miR-211 is reported to be necessary for temporal regulation of *chop* expression (17). Therefore, maintenance of miR-211 would inhibit maximal accumulation of *chop* and this would inhibit the execution of damaged cells. However, validating this model requires further experimental confirmation. 

miR-211 has also recently been implicated in neoplastic growth of colorectal tumors via regulation of the CHD5 tumor suppressor (21). This observation of potential oncomir activity of miR-211 is in contrast with our finding of a tumor suppressor role for miR-211 in esophagus cancer. These findings might support different tumor suppressive and oncomeric activities of miR-211 in different cancers. 

## Conclusion

We show that human miR-211 has a regulatory role on *SOX2OT* and *SOX2* expression in NT2 pluripotent cells. As *SOX2OT* and *SOX2* have already been shown to contribute to tumorigenesis, miR-211 may also have a role but an inhibitory one in the same process. 
